# Morphological Study of Chitosan/Poly (Vinyl Alcohol) Nanofibers Prepared by Electrospinning, Collected on Reticulated Vitreous Carbon

**DOI:** 10.3390/ijms19061718

**Published:** 2018-06-09

**Authors:** Diana Isela Sanchez-Alvarado, Javier Guzmán-Pantoja, Ulises Páramo-García, Alfredo Maciel-Cerda, Reinaldo David Martínez-Orozco, Ricardo Vera-Graziano

**Affiliations:** 1 Tecnológico Nacional de México/Instituto Tecnológico de Cd. Madero, Centro de Investigación en Petroquímica, Prol. Bahía de Aldhair y Av. De las Bahías, Parque de la Pequeña y Mediana Industria, Altamira, Tamaulipas 89600, Mexico; diana.sanchez@itsna.edu.mx (D.I.S.-A.); rd.martinez.orozco@gmail.com (R.D.M.-O.); 2 Instituto Mexicano del Petróleo, Gerencia de Transformación de Hidrocarburos, Eje Central Lázaro Cárdenas No. 152, Col. San Bartolo Atepehuacán, 07730 Ciudad de México, Mexico; jguzmanp@imp.mx; 3 Instituto de Investigaciones en Materiales, Universidad Nacional Autónoma de México, Circuito Exterior S/N, Circuito de la Investigación Científica, Ciudad Universitaria, 04510 Ciudad de México, Mexico; macielal@unam.mx (A.M.-C.); graziano@unam.mx (R.V.-G.)

**Keywords:** chitosan, PVA, nanofibers, electrospinning

## Abstract

In this work, chitosan (CS)/poly (vinyl alcohol) (PVA) nanofibers were prepared by using the electrospinning method. Different CS concentrations (0.5, 1, 2, and 3 wt %), maintaining the PVA concentration at 8 wt %, were tested. Likewise, the studied electrospinning experimental parameters were: syringe/collector distance, solution flow and voltage. Subsequently, the electrospun fibers were collected on a reticulated vitreous carbon (RVC) support for 0.25, 0.5, 1, 1.5, and 2 h. The morphology and diameter of the CS/PVA nanofibers were characterized by scanning electron microscopy (SEM), finding diameters in the order of 132 and 212 nm; the best results (uniform fibers) were obtained from the solution with 2 wt % of chitosan and a voltage, distance, and flow rate of 16 kV, 20 cm, and 0.13 mL/h, respectively. Afterwards, a treatment with an ethanolic NaOH solution was performed, observing a change in the fiber morphology and a diameter decrease (117 ± 9 nm).

## 1. Introduction

The electrospinning technique is an efficient method for the manufacture of micro- and nanofibers; it is also an efficient and simple method to produce homogenous fibers for various applications. This technique relies on multiple operating parameters such as applied voltage, collector type, temperature, relative humidity, flow rate, etc., as well as on intrinsic solution parameters like molecular weight, polymeric concentration, viscosity, surface tension, electrical conductivity, among others. The electrospinning process uses electrostatic forces to produce polymeric fibers. When high voltage is applied, polymer drops are subjected to instability, forming fibers when the surface tension of the polymer solution drop at the tip of the needle is overcome. A series of parameters must be optimized in order to produce fibers instead of drops [1,2,3].

Chitosan (CS) is a biopolymer derived from chitin, often obtained from the exoskeleton of crustaceans. It has very useful properties such as biocompatibility, biodegradability, and antimicrobial activity; however, the production of fibers of this polymer is very difficult due to its insolubility in organic solvents, its ionic character in solution and the formation of three-dimensional networks by strong hydrogen bonds [4,5,6].

In this sense, Li and Hsieh (2006) found that by combining an anionic polymer such as poly (vinyl alcohol) (PVA) and chitosan, which is a cationic polymer, the formation of fibers was improved, which is in contrast with the combination of polymers having the same charge [7]. PVA is a biodegradable polymer that may be used to immobilize chitosan [8]. Moreover, PVA can reduce the crystallinity of chitosan [9]. The functional groups of chitosan make hydrogen bonds with PVA which lead to form defect-free nanofibers [10].

Although CS and PVA can improve the production of fibers, the right proportion to have a stable solution has to be established. In this sense, several works studying different CS/PVA ratios have been reported [11,12,13]. To do so, the calculation of the thermodynamic interaction parameter (X) by using the Flory–Huggins theory is necessary. This parameter represents the energy associated with the blending of polymers; depending on its value, the interaction can be classified as favorable or unfavorable.In our case, we did not consider its calculation because the CS/PVA ratio selected to carry out the electrospinning experiments produced a homogeneous solution, which we could witness in the lab by observing a miscible behavior.

Paipitak et al. [14] used the electrospinning method to produce CS/PVA nanofibers, with a glass plate as collector, and study the effect of CS concentration on the formation of nanofibers. The morphology and diameter of the CS/PVA nanocomposite fibers were analyzed by SEM, finding a fiber diameter of 100 nm. The best CS solution concentration for fiber formation was 2 wt % due to the maximum fiber yield.

According to the points described above and in order to improve the efficiency of the electrospinning method and morphology of the obtained CS/PVA nanofibers, in the present work, reticulated vitreous carbon (RVC) was used as a collector due to its high electrical conductivity and minimal reactivity. RVC is currently used in electrochemical systems such as battery and fuel cells, metal ion removal, electroanalytical analysis, sensors and synthesis of organic compounds. As it can be seen, these applications are related to the promotion of turbulence aimed at increasing the mass transfer phenomena in reactors. To the best of our knowledge, in the present research work, RVC has been used for the first time as a fiber collector in the electrospinning process. The ultimate goal, after fundamental studies, is to produce CS/PVA nanofibers, establishing the best parameters of the electrospinning technique (voltage, drop-collector distance and volumetric flow), on a highly porous support to generate turbulence and adequate contact between the materials and a solution. The obtained results encouraged us to consider the experimental procedure featured in this work for its application in the reduction of sulfur compounds in FCC (Fluid Catalytic Cracking) gasoline, where the chitosan fibers could work as adsorbents of metals such as nickel and molybdenum in an oxidative desulfurization process. The results of this study will be part of a forthcoming paper.

## 2. Results and Discussions

### 2.1. Electrospinning Method: Experimental Parameters

Preliminary electrospinning processes were carried out using different operation parameters. Not all the analyzed electrospinning parameters showed good results since in some cases, the fibers were not uniform with large nodules incorporated into them or simply no fibers were observed.

The operation parameters used to produce fibers are shown in Table 1. In order to establish the best CS/PVA proportions, four of them were experimentally tested (0.5 wt % CS/8 wt % PVA, 1 wt % CS/8 wt % PVA, 2 wt % CS/8 wt % PVA, and 3 wt % CS/8 wt % PVA). As the most uniform nanofibers were obtained with 2 wt % CS/8 wt % PVA, the other three concentrations were discarded (data not shown).Then, the optimal electrospinning conditions were obtained with a 2 wt % CS/8 wt % PVA solution, and a flow rate, distance, and voltage of 0.13 mL/h, 20 cm, and 16 kV, respectively.

**Figure 1:**
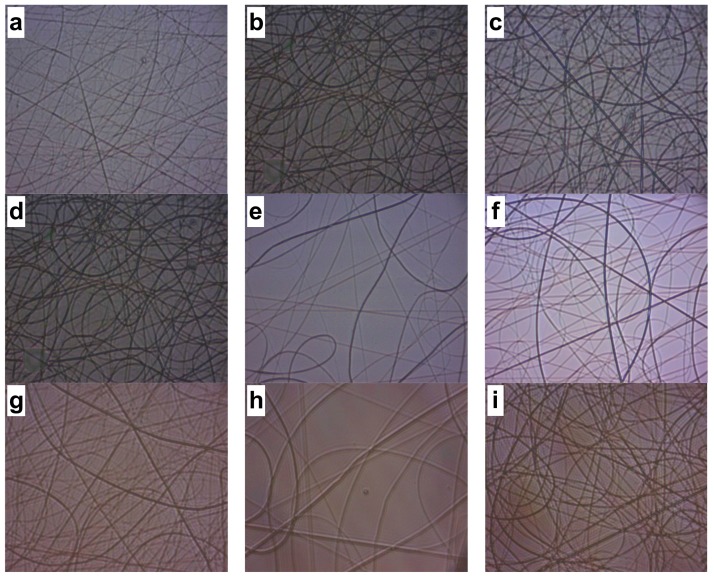

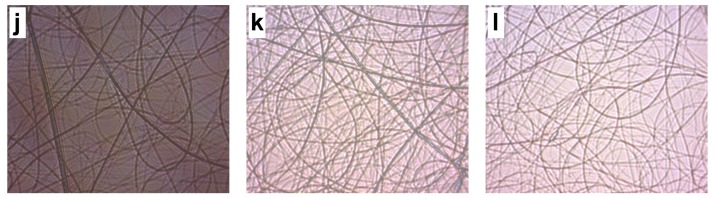
Micrographs of fibers produced by using a 2 wt % CS/8 wt % PVA solution (40× resolution): (**a**) 0.12 mL/h, 15 cm and 14 kV; (**b**) 0.13 mL/h, 15 cm and 14 kV; (**c**) 0.13 mL/h, 15 cm and 15 kV; (**d**) 0.13 mL/h, 15 cm and 14 kV; (**e**) 0.12 mL/h, 20 cm and 14 kV; (**f**) 0.12 mL/h, 25 cm and 14 kV; (**g**) 0.13 mL/h, 20 cm and 16 kV; (**h**) 0.12 mL/h, 25 cm and 16 kV; (**i**) 0.13 mL/h, 20 cm and 14 kV; (**j**) 0.13 mL/h, 25 cm and 14 kV; (**k**) 0.13 mL/h, 20 cm and 16 kV; (**l**) 0.13 mL/h, 25 cm and 16 kV.

Figure 1 shows the CS/PVA electrospun sample using a 2 wt % CS/8 wt % PVA solution, with the parameters featured in Table 1, varying flow rate, distance, and voltage during the performance of the electrospinning technique, where it is observed that the fibers are non-uniform: some fibers are thinner than others and some nodules can be seen, unlike what is observed under the optimal conditions mentioned above. These experiments were performed on a glass surface.

**Figure 2:**
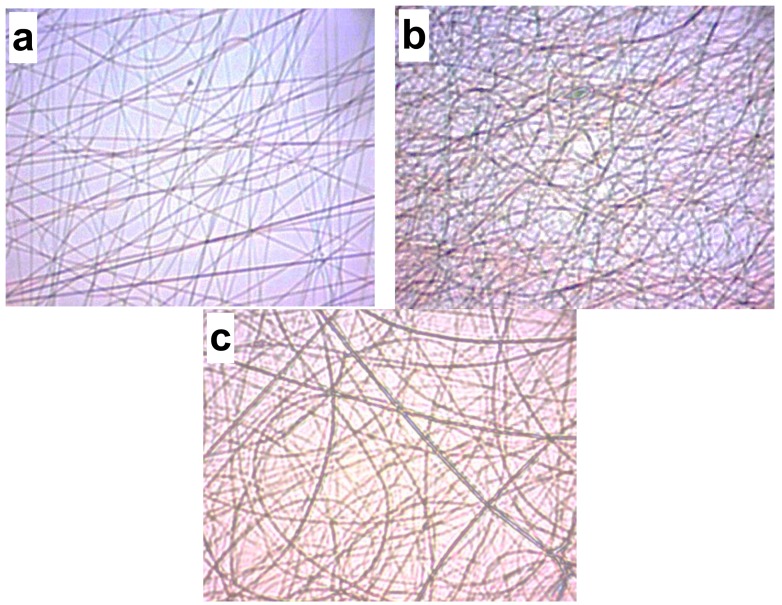
Micrographs of fibers produced with different CS concentrations (wt %): (**a**) 0.5; (**b**) 1; and (**c**) 2 with a voltage, distance, and flow rate of 0.13 mL/h, 20 cm, and 16 kV, respectively (40× resolution).

Figure 2 shows a comparison of optical images for threedifferent solution compositions with optimal conditions. Non-uniform fibers are observed for 0.5 (a) and 0.1 wt % (b) of CS. For 2 wt % CS (c), uniform fibers were obtained.

### 2.2. Electrospun CS/PVA Samples Collected on Reticulated Vitreous Carbon

**Figure 3:**
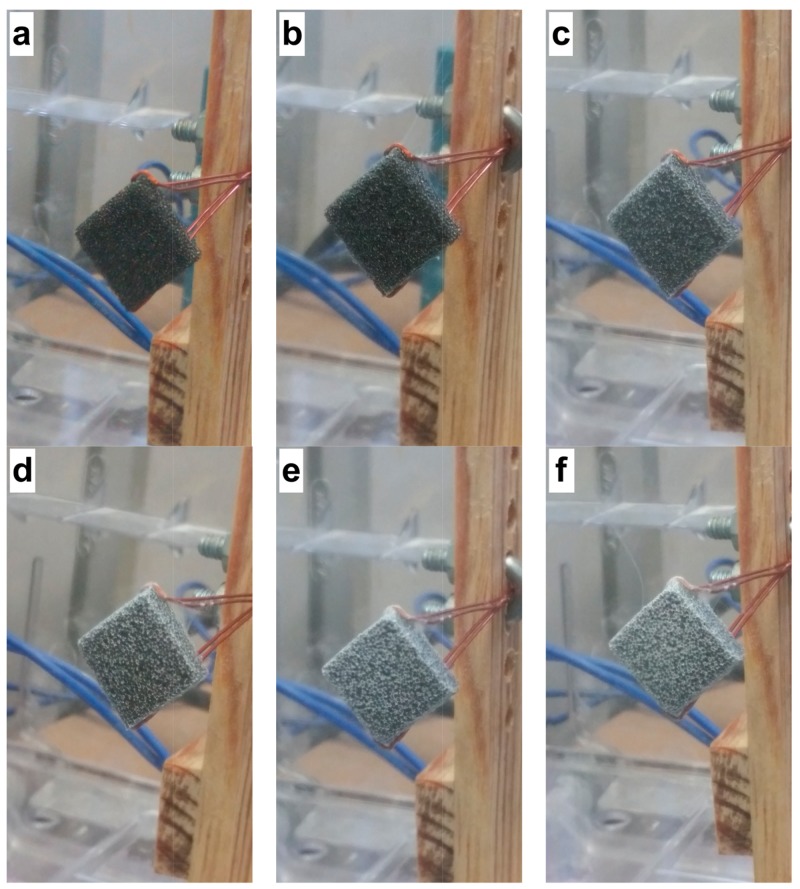
Electrospun fibers on the RVC support: (**a**) 0; (**b**) 15; (**c**) 30; (**d**) 60; (**e**) 90; and (**f**) 120 min.

Figure 3 shows the images of the electrospun fibers on the reticulated vitreous carbon (RVC) support at different times. The deposition of the fibers was observed during the spinning process, and the surface of the RVC support was modified by showing a white coloration caused by the electrospun fibers.

Zargarian and Haddadi [15] indicated that the electrospinning technique can produce fibers with diameters ranging from 5 nm to 1 μm under a high-voltage electrostatic field that runs between a metal capillary syringe and an electrically grounded collector. Fibers made of polymer blends can be produced by this technique. In their study, the authors fabricated nanofibrous scaffolds of poly (ε-caprolactone)/hydroxyapatite/chitosan–PVA in order to investigate the influence of several parameters that affect the process and morphology such as concentration, solvent composition, voltage and tip-to-collector distance (TCD). In this work, the surface morphology of the nanofibrous material was studied using SEM micrographs. The distribution of fiber diameter frequencies was plotted to facilitate the optimization process. The voltage played an important role, while the TCD was largely responsible for the determination of the average diameter of the fibers.

### 2.3. Morphology of the RVC Support and CS/PVA Fibers on RVC

**Figure 4:**
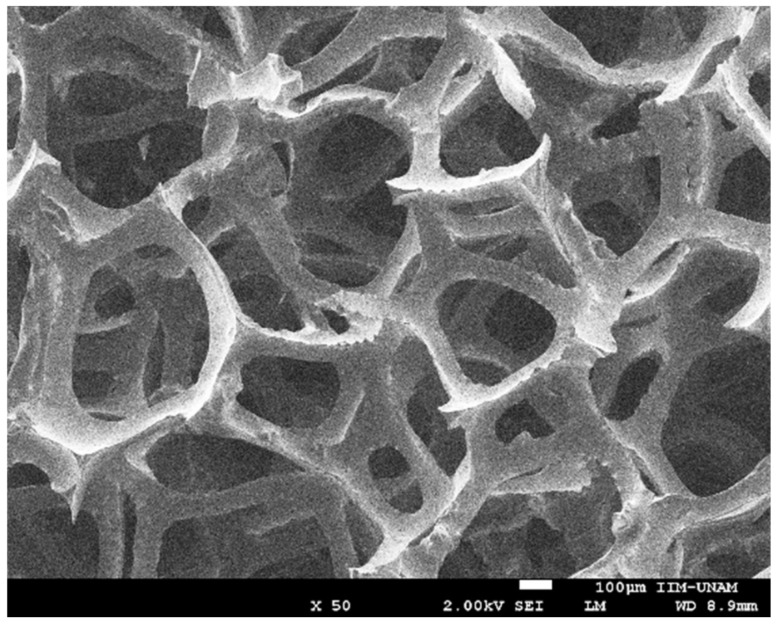
SEM micrograph of reticulated vitreous carbon used as support material.

As for RVC, it is a macroporous polymeric carbon form as it can be seen in Figure 4. This material is obtained, for example, by carbonization of an expanded polymer or a vacuum removed material [16,17]. This material is a solid foam constituted by an open cell network composed of vitreous carbon, a material with high electrical and thermal conductivity. The adjective reticulated means “constructed, arranged or marked as a network or part of a network”. RVC has a remarkably high vacuum volume and surface area. Its rigid structure has low density and withstands low temperatures. Figure 4 shows a SEM micrograph of unmodified RVC.

**Figure 5:**
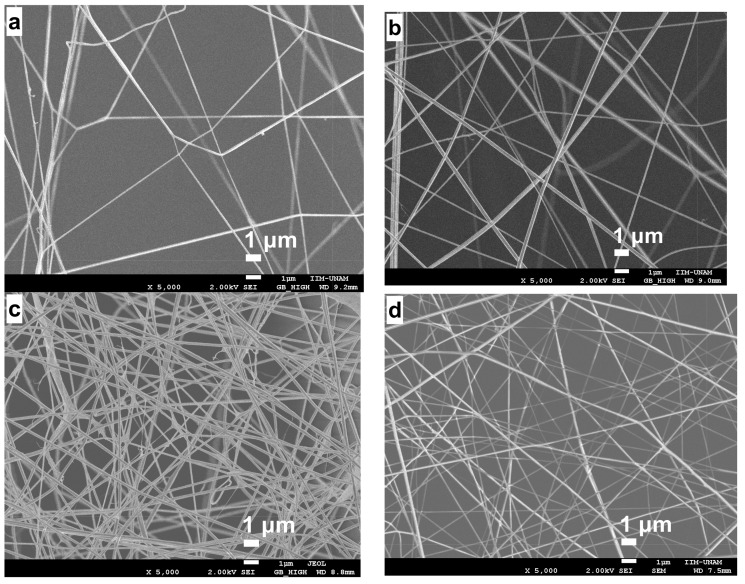

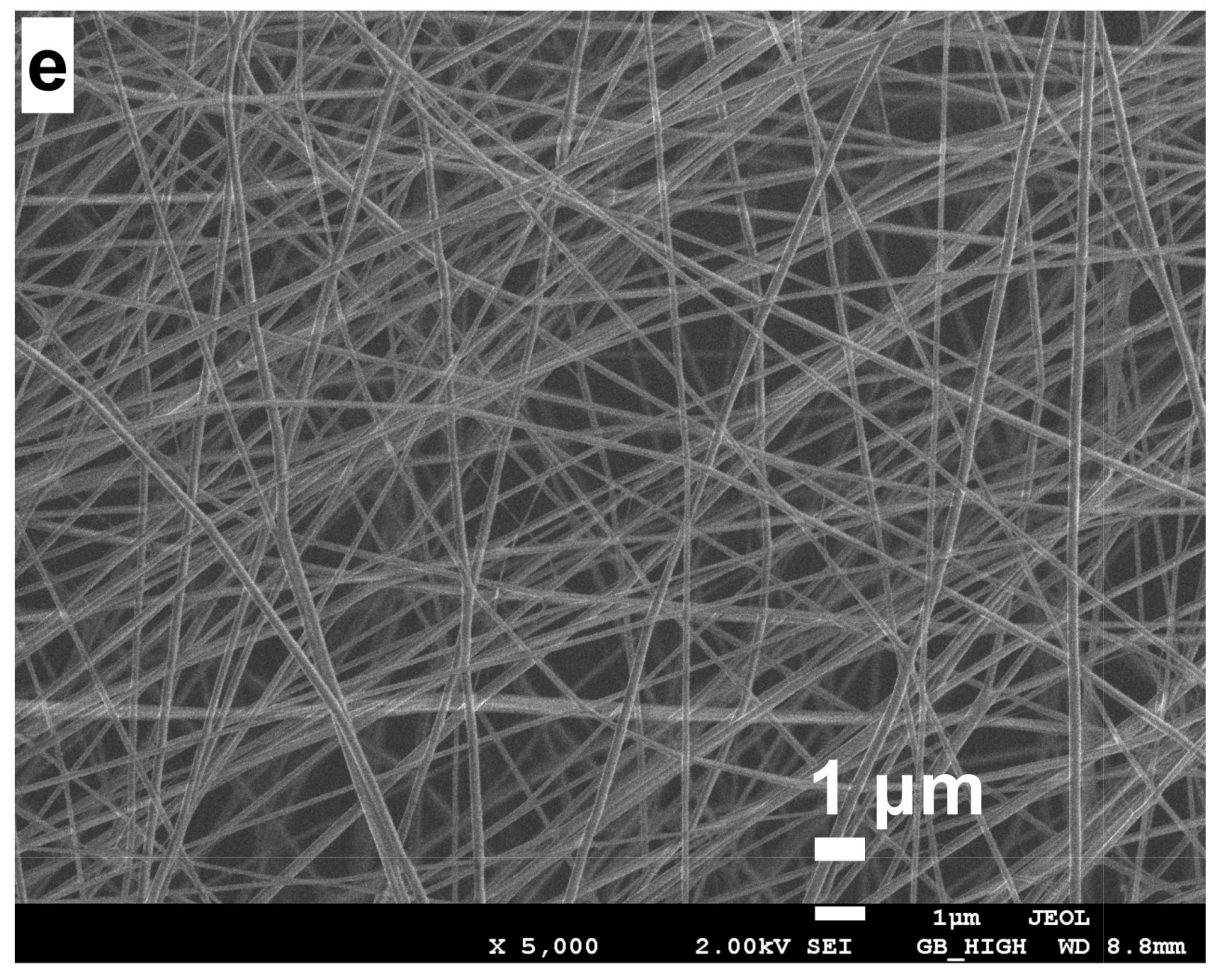
SEM characterization of electrospun nanofibers on a RVC support at different times: (**a**) 15; (**b**) 30; (**c**) 60; (**d**) 90; and (**e**) 120 min.

Figure 5 shows the micrographs of the electrospun fibers on RVC at different times with 2 wt % CS/8 wt % PVA, with a voltage, distance, and flow rate of 0.13 mL/h, 20 cm, and 16 kV, respectively. It is observed that the deposited fibers increased as the electrospinning time increased too; very few defects and a relatively uniform morphology can be observed.

**Figure 6:**
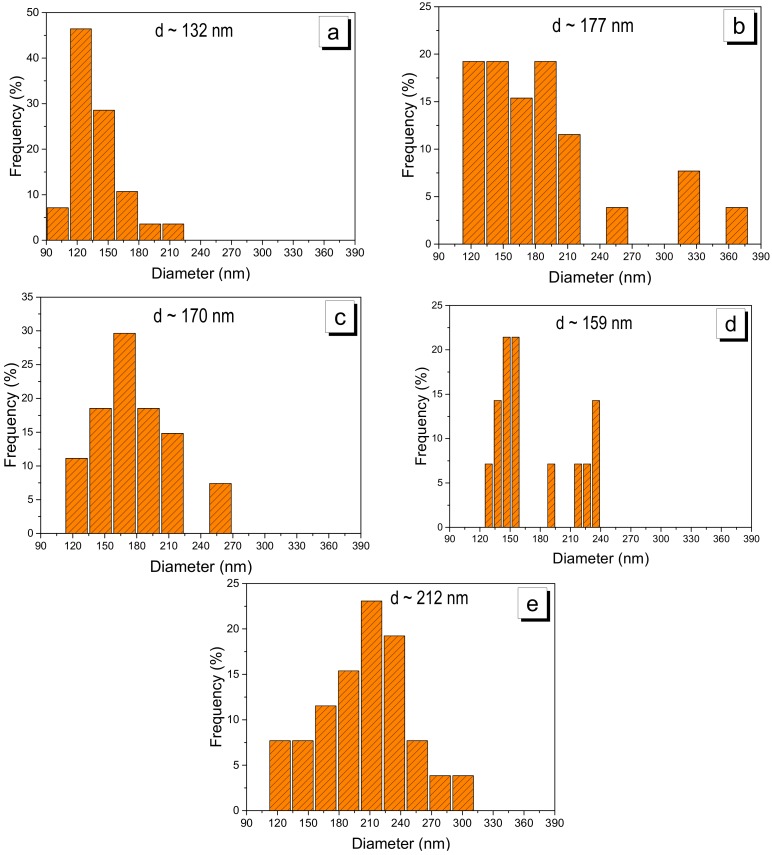
Histograms of nanofibers on the RVC support: (**a**) 15; (**b**) 30; (**c**) 60; (**d**) 90; and (**e**) 120 min.

For the diameter frequency histograms, about 100 electrospun fibers were analyzed with particle counting. Figure 6 presents the histograms of the nanofibers analyzed above, where diameters from 90 to 390 nm were observed.

Table 2 shows the average fiber diameters at electrospinning times of 15 min, 0.5, 1, 1.5 and 2 h. Diameters from 132 to 212 nm were observed with a standard deviation from ±6 to ±20 nm.

### 2.4. Stabilization of CS/PVA Nanofibers

**Figure 7:**
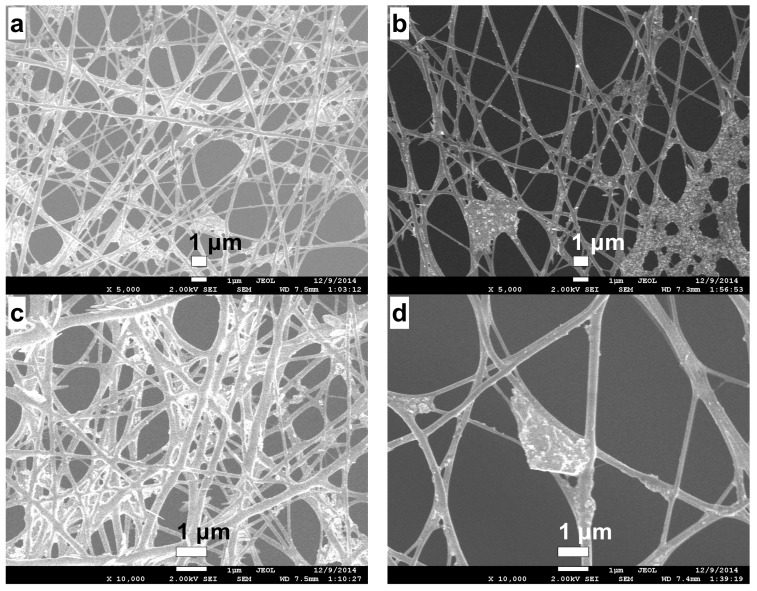
SEM micrographs of CS/PVA electrospun nanofibers deposited on RVC for 90 min before (**a**,**c**) and after (**b**,**d**) ethanolic NaOH solution treatment.

Figure 7 shows micrographs of CS/PVA nanofibers deposited on RVC at an electrospinning time of 90 min before ((a) and (c)) and after ((b) and (d)) the ethanolic NaOH solution treatment used to stabilize the nanofibers. This treatment resulted in a fiber diameter decrease, which is in good agreement with results reported in the literature [18].

Table 3 presents the diameter of the electrospun fibers for 90 min before and after the alkaline treatment, which produced a diameter decrease of 44 nm.

### 2.5. Solution Viscosity

**Figure 8:**
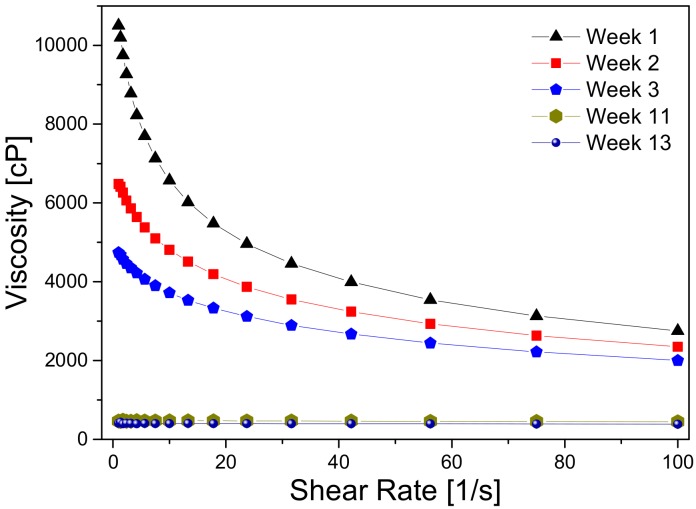
Viscosity variation of the 2 wt % CS/8 wt % PVA solution.

The viscosity of the CS/PVA solution decreased with time (weeks) from 10,500 to 410 cP, which was due to the hydrolysis of chitosan, as stated elsewhere [19,20]. Based on this fact, the electrospun nanofibers were produced by using a recently prepared solution. The results are shown in Figure 8.

**Figure 9:**
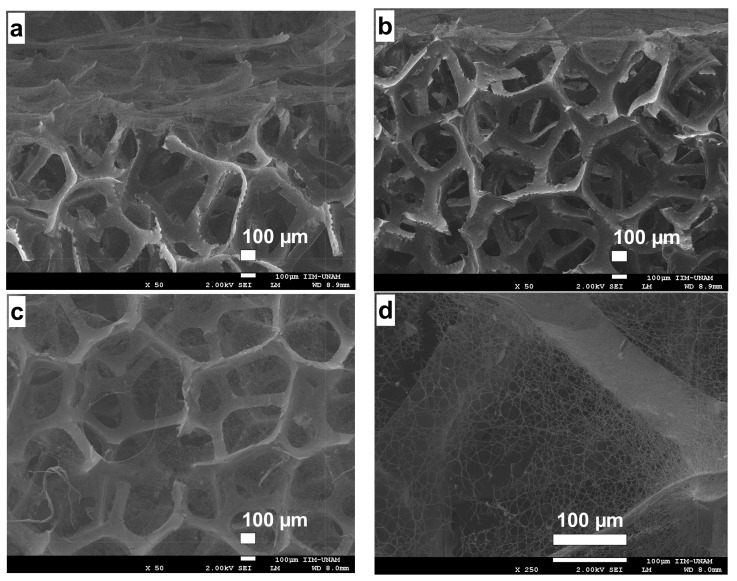
SEM micrographs of electrospun nanofibers deposited on RVC (90 min): (**a**,**b**) cross sections; (**c**,**d**) top-view sections.

Figure 9 shows the micrographs obtained from the electrospun process on RVC, where cross ((a) and (b)) and topview ((c) and (d)) sections were taken for the SEM analysis. It is observed that the fibers are only found on the RVC surface, confirming the fact that they did not penetrate into the pores of the RVC support.

## 3. Materials and Methods

### 3.1. Materials

CS from crab shells with deacetylation degree of 85% (lot number 91k1265) was used as received from Sigma Aldrich. Medium molecular weight PVA was purchased from Sigma Aldrich and used as received. Acetic acid (99.7%) was acquired from J.T Baker. RVC 60 ppi (normal pores per lineal inch) was obtained from Electrosynthesis, Co. Inc., Lancaster, NY, USA.

### 3.2. Preparation of the CS/PVA Solution

The electrospinning solutions were prepared by dissolving a maximum of 3 wt % of CS in acetic acid at 2 wt %. This solution was magnetically stirred until complete dissolution at room temperature. PVA powder was dissolved in deionized water at 8 wt % near its boiling point to facilitate complete dissolution. Subsequently, both solutions were homogenized by magnetic stirring.

In order to study the component ratio effect on the electrospinning process, the CS concentration was varied using 0.5, 1, 2, and 3 wt % while the PVA concentration remained at 8 wt % for each prepared solution.

### 3.3. Electrospinning Process

Electrospinning solutions were pumped using a 5 mL commercial plastic syringe and a Programmable Syringe Pump NE-1600. The studied flow rates were adjusted to 0.10, 0.12, 0.13, 0.15, and 0.20 mL/h at three different working voltages: 14, 15, and 16 kV, controlled by a high DC (Direct Current) voltage supply (Glassman High Voltage EH60). A glass plate or RVC was used as a collector, and the tip-to-collector distanceswere set at 15, 20, and 25 cm. All electrospinning procedures were carried out at room temperature (i.e., at around 23 °C). As the solution viscosity is a very important parameter, all the electrospinning experiments were carried out with recently prepared CS/PVA solutions (no more than 1 week old).

### 3.4. Alkaline Treatment with NaOH

The as-obtained electrospun nanofibers were immersed overnight in a saturated NaOH ethanolic solution in order to analyze the alkaline treatment effect. The treated samples were then rinsed with water and dried in air to be observed by SEM.

### 3.5. Viscometric Studies

The solution viscosity was measured in a rheometer (MCR Physica 301, Anton Paar) with cone and plate geometry, varying the shear rate between 1 and 100 rad/s. The measurements were carried out after 1, 2, 3, 11, and 13 weeks of their preparation. The storage temperature was 25 °C.

## 4. Conclusions

In the present study, CS/PVA nanofibers were manufactured by the electrospinning technique. The use of RVC allowed the formation of homogeneous fibers, compared to the fibers collected on a glass plate, with diameters ranging from 132 to 212 nm, making RVC a suitable substrate to obtain thin fibers. However, the deposit of fibers occurred only on the substrate surface. After testing CS concentrations of 0.5, 1, 2, and 3 wt %, it was found that the best solution to produce fibers was that containing 2 wt % CS and 8 wt % PVA. After evaluating electrospinning parameters such as flow rate, tip-to-collector distance, and voltage on either glass or RVC substrates, the optimum parameters for producing CS/PVA electrospun nanofibers were a voltage, distance, and flow rate of 16 kV, 20 cm, and 0.13 mL/h, respectively. , which allowed the manufacture of uniform fibers that were observed by SEM. The use of RVC improved the formation of homogeneous fibers compared to the fibers collected on the glass plate, with diameters ranging from 132 to 212 nm. In order to obtain chemically stable CS/PVA nanofibers, it was necessary to treat the nanofibers with an ethanolic NaOH solution.

## Figures and Tables

**Table 1:** Experimentally evaluated parameters using a 2 wt % CS/8 wt % PVA solution.

2 wt. CS/8 wt % PVA Solution	Image	Flow Rate	Distance	Voltage	Observations *
	Id.	mL/h	cm	kV	
Flow rate	a	0.12	15	14	Non-uniform fibers
	b	0.13	15	14	Non-uniform fibers with nodules
Voltage	c	0.13	15	15	Non-uniform fibers with nodules
	d	0.13	15	14	Non-uniform fibers
Tip–target distance	e	0.12	20	14	Non-uniform fibers
	f	0.12	25	14	Non-uniform fibers
	g	0.13	20	16	Non-uniform fibers
	h	0.12	25	16	Non-uniform fibers
	i	0.13	20	14	Non-uniform fibers
	j	0.13	25	14	Non-uniform fibers
	k	0.13	20	16	Uniform fibers
	l	0.13	25	16	Non-uniform fibers with nodules

* Uniformity criteria were the fiber abundance and lack of nodules observed in the micrographs shown in Figure 1. CS: chitosan; PVA: poly (vinyl alcohol).

**Table 2:** Diameters of the electrospun nanofibers.

Parameter	15 min	30 min	1 h	1.5 h	2 h
Diameter (nm)	132	177	170	159	212
Standard deviation (nm)	±20	±9	±7	±11	±6

**Table 3:** Diameters of the electrospun fibers before and after the alkaline treatment.

Parameter	Before	After
Diameter (nm)	159 ± 11	115 ± 9
